# Nachhaltiges Wachstum: Braucht Deutschland ein Investitionsprogramm?

**DOI:** 10.1007/s10273-021-2865-x

**Published:** 2021-06-23

**Authors:** ZG Überblick

**Keywords:** E62, H12, R53

## Abstract

Der Wirtschaftseinbruch bedingt durch die Corona-Pandemie trifft in Deutschland auf einen Strukturwandel, der speziell durch die notwendige Digitalisierung und Dekarbonisierung sowie den demografischen Wandel getrieben ist. Einerseits wird kurzfristig durch das Corona-Konjunkturpaket und den EU-Aufbaufonds auf die Krise reagiert. Andererseits werden eine seit längerem existierende Investitionslücke sowie Hemmnisse hinsichtlich der Abrufung vorhandener Mittel bemängelt. Die Autor:innen diskutieren, welche fiskalischen Spielräume vorhanden sind, sowie auf welche Weise und in welche Bereiche öffentliche Investitionen in Deutschland getätigt werden müssten, um nachhaltiges Wirtschaftswachstum zu ermöglichen.

## Abstract (en)

The economic slump caused by the COVID-19 pandemic is accompanied by structural change in Germany, driven in particular by the need for digital transformation and decarbonization as well as demographic change. On the one hand, the coronavirus economic stimulus package and the EU recovery fund are responding to the crisis in the short term. On the other hand, the authors criticize a long-standing investment gap and the obstacles to the use of existing funds. The authors discuss what fiscal leeway is available and in which ways and in which areas public investment would have to be made in Germany in order to enable sustainable economic growth.

